# Dynamics of a Class 1 Integron Located on Plasmid or Chromosome in Two *Aeromonas* spp. Strains

**DOI:** 10.3389/fmicb.2016.01556

**Published:** 2016-09-28

**Authors:** Abigail Pérez-Valdespino, Alfredo Lazarini-Martínez, Alejandro X. Rivera-González, Normand García-Hernández, Everardo Curiel-Quesada

**Affiliations:** ^1^Department of Biochemistry, Escuela Nacional de Ciencias Biológicas, del Instituto Politécnico NacionalMexico City, Mexico; ^2^Molecular Genetics Laboratory, Medical Research Unit, Pediatric Hospital, Centro Médico Nacional Siglo XXI, Instituto Mexicano del Seguro SocialMexico City, Mexico

**Keywords:** *Aeromonas*, Class 1 integrons, streptomycin, integrase expression, SOS response

## Abstract

Integrons are non-mobile bacterial genetic elements that carry different cassettes conferring antibiotic resistance. Cassettes can excise or integrate by action of an integron-encoded integrase, enabling bacteria to face environmental challenges. In this work, the functionality and dynamics of two integrons carrying the same cassette arrangement (*dfr*A12–*orf*F–*aad*A2), but located on plasmid or chromosome in two different strains were studied. In order to demonstrate the functionality of the Class 1 integrase, circular cassette integration intermediaries were PCR amplified by PCR using extrachromosomal DNA extracted from bacteria grown in the presence or absence of cassette-encoded antibiotics. Circular *aad*A2 and *dfr*A12–*orf*F–*aad*A2 cassettes were detected in cultures grown either in the presence or absence of antibiotics in both strains. No *dfr*A12–*orf*F circular intermediates could be detected under any culture conditions. These results show that both integrons are functional. However, these elements show different dynamics and functionality since the presence of streptomycin led to detectable gene rearrangements in the variable region only in the strain with the plasmid-born integron. In addition, complete integration products were demonstrated using a receptor molecule carrying an empty integron. In this case, integration products were observed in both strains even in the absence of antibiotics, but they were more evident in the strain with the plasmid-located integron when streptomycin was present in the culture medium. This suggests that integrons in the two strains respond differently to streptomycin even though DNA sequences upstream the *int*I1 gene, including the l*exA* boxes of both integrons are identical.

## Introduction

Bacteria from the genus *Aeromonas* contain a variety of genetic elements including plasmids, transposons, bacteriophages, and integrons that contribute to the spread of antibiotic resistance determinants (Piotrowska and Popowska, 2015). Previous studies have found integrons in *Aeromonas* predominantly belonging to Class 1-carrying gene cassettes conferring resistance to several antibiotic groups. The most often found are different aminoglycoside resistance genes (*aad*A family) and trimethoprim-resistance genes (*dfr* family; Deng et al., 2016).

Integrons are genetic platforms that allow bacteria to acquire, excise, and reorder gene cassettes, which enables them to face environmental stress (Escudero et al., 2015). Integrons are unable to move by themselves. Platform components are the *int*I gene encoding a site-specific tyrosine-recombinase (IntI), which catalyzes the excision and integration of gene elements frequently encoding antibiotic resistance known as cassettes. A primary recombination *att*I site is found upstream the *int*I gene. Between these two elements a region carrying two divergent promoters is found. One of these promoters (P*int*I) controls the *int*I gene expression, whereas the Pc promoter is responsible for the expression of gene cassettes located in the variable region, which generally lack a promoter of their own (Mazel, 2006). Cassettes in the variable region are spaced by *att*C sequences, which are recognized by the integrase IntI to be excised or integrated. Excised genes move to other positions in the variable region via covalently closed circular intermediaries (Partridge et al., 2009). This cassette shuffling has a crucial effect on gene expression since genes closer to Pc are usually more expressed than those located farther away. Cassette rearrangements depend on the integrase activity, which is often regulated by the SOS response. Several antibiotic families are able to trigger this type of response, allowing bacteria to adapt to different selective pressures (Strugeon et al., 2016).

Integrons are classified in two groups depending on their location: mobile integrons (MIs), intimately associated to plasmids and transposons, which are the main vehicles of multiresistance in bacteria (Stalder et al., 2012), or chromosomal integrons (CIs), which are large and sedentary elements compared to mobile integrons. Superintegrons are a subgroup of CIs whose variable region exhibits more than 20 gene cassettes (Rowe-Magnus et al., 1999).

In this work, we aimed to study the dynamics and functionality of two identical Class 1 integrons with the cassette arrangement *dfr*A12–*orf*F–*aad*A2, found in *Aeromonas dhakensis* 3430-1 and *A. hydrophila* 6479 and differing in their localization (chromosome versus plasmid, respectively; Pérez-Valdespino et al., 2009).

## Materials and Methods

### Bacterial Strains and Plasmid

The two *Aeromonas* strains analyzed in this study were isolated from stool samples of patients who attended the Public Health Service at Hidalgo State, Mexico in 2005. The samples were provided by the patients in order to establish a diagnostic, and were not obtained directly from the patients themselves. Therefore, informed written consent was not necessary for this study. Strains were assigned to the *Aeromonas* genus through amplification of the GCAT (glycerophospholipid cholesterol acyl transferase) gene. For species confirmation the *rpo*D (RNA polymerase σ70 factor) gene was PCR amplified and sequenced (Figueras et al., 2009; Beaz-Hidalgo et al., 2010). The same Class 1 integron with different localization (chromosomal in strain 3430-1 and plasmid in strain 6479) was studied (Pérez-Valdespino et al., 2009). Acceptor pICV8 plasmid contains an *att*I site for cassette capture, an interrupted integrase gene, a Pc promoter, an incomplete *aad*A1 gene, and a zeocin-resistance gene (Shearer and Summers, 2009). This plasmid was transformed in *Escherichia coli* S17-1 λ*pir* for mating experiments.

### Determination of Minimal Inhibitory Concentrations

The minimum inhibitory concentration (MIC) for two antibiotics (trimethoprim and streptomycin) was established by the broth macrodilution method (Wikler, 2007).

### Isolation of Circular Covalently Closed Integration Intermediaries

Extrachromosomal DNA was isolated using the Wizard Plasmid Miniprep Kit (Promega, Fitchburg, WI, USA) from overnight cultures grown in the presence or absence of antibiotics. After extraction, the recovered 50 μL extrachromosomal DNA was concentrated 10 times in order to increase the concentration of the integration intermediaries.

### PCR Amplification of Circular Integration Intermediaries

Extrachromosomal DNA (1 μL) was used as a template to search for circular integration intermediaries through polymerase chain reaction. For this purpose, outward primers (aadA2 amino, aadA2 carboxy, dfr12 amino, dfr12 carboxy, and orfF carboxy), aimed to amplify single, double or triple excision intermediaries were designed (**Table 1**). Amplifications were performed in an Eppendorf Mastercycler^®^ gradient thermal cycler (Eppendorf, Hamburg, Germany). Reactions included 12.5 μL PCR Master Mix 2X (Fermentas, Vilnius, Lithuania), 3 μL (200–300 ng) template DNA from cultures grown with or without antibiotic and 10 pmol/μL of each primer to a final volume of 25 μL. Amplification consisted of DNA denaturation at 94°C for 5 min followed by 30 PCR cycles (DNA denaturation at 94°C for 0.5 min, primer annealing at 53°C for 0.5 min, and DNA extension at 72°C for 1.0 min), and a final extension step at 72°C for 7 min. Amplicons were reamplified and sequenced to confirm the identity of PCR products.

**Table 1:** Sequence of primers used in this study.

Primer name	Sequence 5′–3′	Template	Reference
aadA2 amino	GCCATCCACTGCGGAG	Circular excision	This study
aadA2 carboxy	GCCCGTCTTACTTGAAGC	Intermediaries	
dfr12 amino	TTTCCCTCAGTGAGTCTGC		
dfr12 carboxy	ATACACTCTGGCACTACCTCAC		
orfF carboxy	GCTTACCTCGCCCGTTAG		
IntPcFw	AACCCAGTGGACATAAGCC	*lex*A box	
dfr12 amino Q	CGTACTGATTCCGAGTTCAT		
in-F	GGCATCCAAGCAGCAAGC	Integron variable	Schmidt et al., 2001
in-B	AAGCAGACTTGACCTGAT		
8E-U1	CCTCGTTλGGACAAGGACCTGAG	pICV8 derivatives	Shearer and Summers, 2009
aadA2-L2	GCGAGCTGCAATTTGGAGAATGG		
4E-L2	GCCTATGCCTACAGCATCCAGGGTGAC		
dfrA12 U1	GCCTGλAGCTλTGCCGTTTG		
Int1_up	λCCGAGGATGCGAACCACTT	mRNA	Soler et al., 2004
Int1_dw	CAACCTTGGGCAGCAGCGAA		
GCAT fw	CTCCTGGAATCCCAAGTATCAG		
GCAT rv	GGCAGGTTGAACAGCAGTATCT		

### PCR Amplification of Variable Integron Regions

Cells grown overnight at 37°C in Luria Broth supplemented with streptomycin (32 or 256 μg/mL), trimethoprim (128 μg/mL), or in the absence of antibiotics. DNA was extracted (Sambrook and Russell, 2001) and used to amplify the integron variable regions. Amplifications were performed with the previously reported primers in-F and in-B (Schmidt et al., 2001). PCR conditions were as above, except the annealing temperature was 56°C and the extension time was 1.5 min.

### Assay to Assess the Mobility of Cassettes from Chromosome or Plasmid

Mating experiments using *E. coli* S17-1 λ *pir* (PICV8) as a donor and strains 3430-1 and 6479 as recipients were performed to assess gene cassette mobility. Transconjugants were selected on LB plates containing 50 μg/mL ampicillin plus 75 μg/mL zeocin. After selection strains were grown in antibiotic-free medium and plasmid DNA was isolated in order to confirm the presence of pICV8. Plasmids extracted from transconjugants grown overnight in the presence of 32 and 256 μg/mL streptomycin or in absence of antibiotic were used as templates for PCR amplification reactions. Primers to amplify the pICV8/cassette junctions (Shearer and Summers, 2009 and this work) were used for these experiments (**Table 1**).

### Real Time RT-PCR

*int*I1 expression levels were evaluated through real-time RT-PCR from cultures grown in the presence of 32 or 256 μg/mL streptomycin or in the absence of antibiotics. Total RNA from cultured cells was extracted using the RNeasy Mini Kit (Qiagen, Valencia, CA, USA). RNA was reverse transcribed and amplified using the SuperScript^TM^ III Platinum^®^ SYBR^®^ Green One-Step qRT-PCR Kit, supplemented with ROX dye (Life Technologies, Carlsbad, CA, USA). Each experimental condition was repeated three times in assays performed in triplicate. Expression values were normalized using the GCAT gene and relative expression levels were calculated using the 2^-ΔΔCT^ method (Yuan et al., 2008). The primers used in real time RT-PCR assays are shown in **Table 1**.

### PCR Amplification of *lex*A Boxes

PCR was performed with primers IntPcFw and dfr12 amino Q that were designed for this work (**Table 1**). The amplification was performed as above. Amplicons were sequenced to analyze the *lex*A boxes.

## Results

### Strains Identification

Strains were assigned to the genus *Aeromonas* through PCR amplification of GCAT gene. Species were assessed by amplification and sequencing of the *rpo*D gene. Strains 3430-1 and 6479 were classified as *A. dhakensis*, previously *A. caviae* (Pérez-Valdespino et al., 2009) and *A. hydrophila*, respectively.

### Determination of Minimal Inhibitory Concentrations

The MIC values for streptomycin and trimethoprim for both strains were 32 μg/mL and 128, respectively.

### Study of Integration Intermediaries through PCR

Circular excision products corresponding to the individual *aad*A2 cassette and to the entire variable region including cassettes *dfr*A12–*orf*F–*aad*A2 were detected in cultures grown either in the presence or absence of antibiotics in both strains (data not shown). Covalently closed circular cassette *dfr*A12–*orf*F was not detected under any experimental conditions. Identity of circular intermediaries was confirmed by sequencing.

### Amplification of the Integron Variable Regions from Cultures Grown in the Presence or Absence of Antibiotics

**FIGURE 1:**
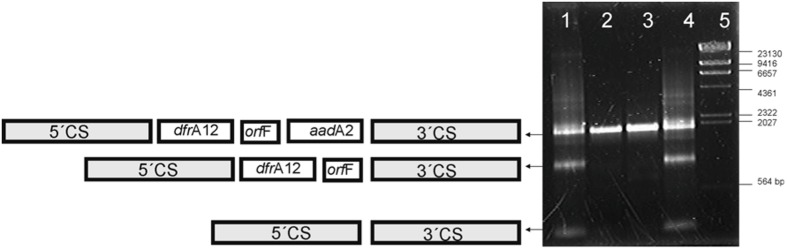
**Amplicons from the variable region of Class 1 integrons.** Lanes 1 and 4, strain 6479 grown in the presence of 32 and 256 μg/mL streptomycin, respectively. Lanes 2 and 3, strain 3430-1 grown in the presence of 32 and 256 μg/mL streptomycin, respectively. Lane 5, λ/HindIII fragments (Fermentas, Vilnius, Lithuania). Diagrams on the left show the integron arrangements documented.

The integron variable regions were amplified from total DNA extracted from cells cultured in medium with or without antibiotics. Changes in the variable region were observed in strain 6479 but no in strain 3430-1 grown in the presence of streptomycin. Trimethoprim did not induce any changes in the integron variable regions. Each amplification band was sequenced to characterize the different arrangements of the variable region (**Figure 1**).

### Assessment of Cassette Mobility from Plasmid or Chromosome DNA to the Acceptor Plasmid

**FIGURE 2:**
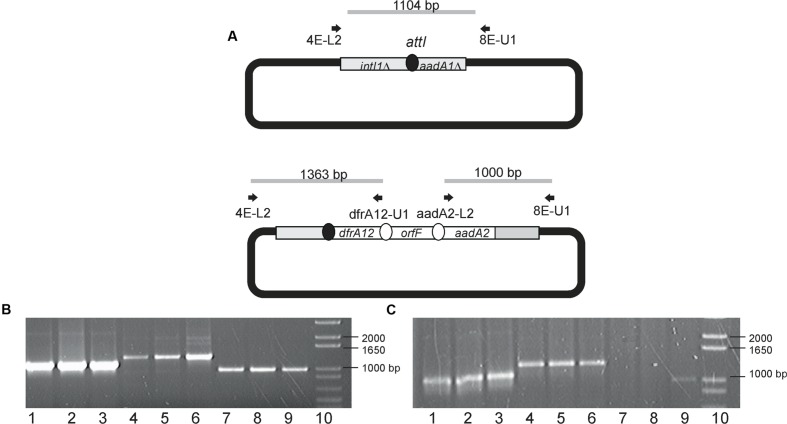
**pICV8 integration products. (A)** Diagram showing primers used to demonstrate cassette insertions in pICV8. **(B,C)** Amplicons derived from pICV8 cassette insertion products in strains 6479 and 3430-1, respectively. Lanes 1–3 were amplified using 4E-L2 and 8E-U1. Lanes 4–6 were amplified using 4E-L2 and drfA12-U1. Lanes 7–9 were amplified using aadA2-L2 and 8E-U1. Lanes 1, 4, and 7, strains grown without streptomycin. Lanes 2, 5, and 8, trains grown with 32 μg/mL streptomycin. Lanes 3, 6, and 9, strains grown with 256 μg/mL streptomycin. Lanes 10, molecular weight 1 Kb plus (Invitrogen, Carlsbad, CA, USA).

In order to substantiate whether Class 1 integron-encoded IntI1 could mediate the excision and subsequent integration of gene cassettes to an empty integron, the acceptor plasmid (pICV8) was introduced by conjugation to both *Aeromonas* strains. After mating, plasmid DNA was extracted from transconjugants and cassettes inserted into pICV8 were amplified by PCR. It was observed that IntI1 was able to mediate the movement of the entire *dfr*A12–*orf*F–*aad*A2 region to pICV8 in *A. hydrophila* 6479 and *A. dhakensis* 3430-1. These cassette movements were observed even in the absence of streptomycin. Streptomycin addition led to the occurrence of two additional integration products in strain 6479 (*aad*A2–*dfrA*12–*orf*F and *orf*F*–aad*A2–*dfr*A12). **Figure 2** shows the amplicons produced as well as the primers employed. Structures of these new arrangements show that integration in pICV8 was accompanied with cassette reorganization. It is important to emphasize that the product from the variable region maintaining the original gene arrangement was always more abundant than those resulting from cassette switching. These integration events were more evident at the highest streptomycin concentration used (**Figure 2**).

### Analysis of Integrase Expression

**FIGURE 3:**
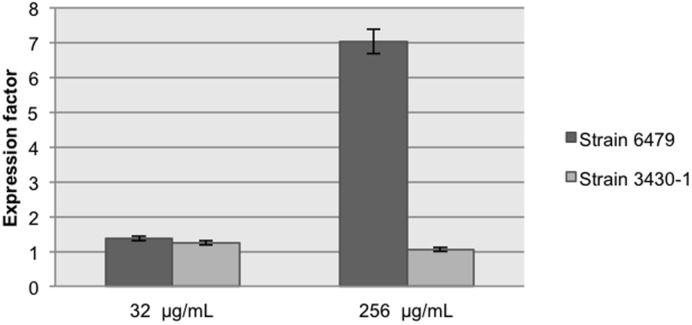
***int*I1 relative expression in the presence of two streptomycin concentrations.** Results are the mean of three independent experiments performed in triplicate.

Quantitative RT-PCR assays evidenced changes in the *int*I1 transcript levels in the presence of 256 μg/mL streptomycin in *A. hydrophila* 6479 (**Figure 3**), resulting in increased cassette rearrangements in this strain (**Figure 2**). No change in integrase mRNA levels could be documented in *A. dhakensis* 3430-1 in the presence of streptomycin.

### Amplification of the *lex*A Box

**FIGURE 4:**
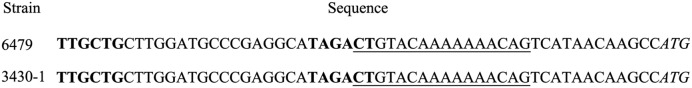
**Sequences upstream of the *int*I1 gene.** The SOS boxes are underlined. Start codons are italicized. -35 and -10 consensus sequences are in bold type.

In order to determine if both integrons are potentially regulated by the SOS response, we amplified a 286-bp long region upstream of the *int*I1 gene. Analysis of the sequences showed identical *lexA* boxes in both strains (**Figure 4**).

## Discussion

Integrons are genetic systems that help prokaryotes adapt to different environments, acting as gene reservoirs located in both mobile and non-mobile genetic elements (Stokes and Gillings, 2011). The presence of integrons in environmental and clinical *Aeromonas* strains has been documented (Chang et al., 2007; Jacobs and Chenia, 2007). No obvious differences in the gene arrangements between these types of strains seem to exist. Literature reports that Class 1 integrons are mainly associated to plasmids, which contributes to their dissemination. It is important to point out that the genus *Aeromonas* has a low incidence of plasmids (Brown et al., 1997; Lee et al., 2008). Additionally, it has been described that some plasmids are unable to establish in some *A. hydrophila* strains (Bello-López et al., 2012). In this work, we aimed to investigate the functionality and dynamics of two Class 1 integrons with the same variable region but different localization (Pérez-Valdespino et al., 2009). The occurrence of identical variable regions in integrons from different strains could indicate that such elements are unable of exchanging cassettes and are thus inactive. This work shows that both integrons are functional, as evidenced by the detection of circular integration intermediaries. We could detect the formation of an *aad*A2 circular cassette and even a circular intermediate corresponding to the entire variable region in both strains. To this end, we used highly concentrated extrachromosomal DNA as a template. Others have reported the excision of single gene cassettes, but only using hosts that overexpress the integrase gene (Collis et al., 2001; Dubois et al., 2007). These data confirm the extraordinary low abundance of circular cassettes. To our knowledge, no other reports detecting circular intermediaries bearing more than one cassette exist.

Selective pressure with streptomycin led also to detectable modifications of the integron variable regions. Changes in the variable region identical to those expected to result from the excision of the circular intermediaries were detected only in the strain carrying the plasmid version of the integron (**Figure 1**). Results reveal that integrons from these two different localizations respond differently to streptomycin. One of the rearrangements lacks the *aad*A2 cassette. Excision and reintegration of the cassette in the first position of the array, closer to the Pc promoter, would increase the streptomycin resistance. The faint band around 2000 bp in **Figure 2B** lane 4–6 could result from this rearrangement. In this way, excision and integration of cassettes would help bacteria to cope with the selective pressure posed by streptomycin (Mazel, 2006; Michael and Labbate, 2010). Literature reports show that trimethoprim, which inhibits DNA synthesis, induces the expression of integrase in *E. coli* and *Vibrio* sp. (Guerin et al., 2009). In contrast, our data show no integrase induction by trimethoprim. Baharoglu et al. (2013) reported that streptomycin induces SOS response in *Vibrio cholerae* but not in *E. coli*. In this work, we demonstrate that this antibiotic acts as an integrase inducer only in strain 6479 (**Figure 1**).

Shearer and Summers (2009) quantified the integrase activity by measuring the integrase-mediated plasmid recombination products. To determine if plasmid or chromosomal localization influence the integrase ability to transfer gene cassettes to a recipient plasmid, we introduced pICV8 to strains 3430-1 and 6479 and searched for integration products. We found that the original cassette array (*dfrA*12–*orf*F–*aad*A2) was successfully integrated into pICV8 regardless of its plasmid or chromosomal origin. We conclude that normal integrase levels are sufficient to complete the cassette integration event. It is also possible that integration was due to increased integrase levels triggered by the entrance of single stranded DNA during conjugation (Baharoglu et al., 2010). Transconjugant *Aeromonas* bearing pICV8::*dfrA*12–*orf*F–*aad*A2 were grown in the presence of streptomycin. Strain 6479 yielded a higher number of arrangements than strain 3430-1, suggesting that IntI1 is more expressed in the first strain (**Figure 2**). This was confirmed by qRT-PCR (**Figure 3**).

Recent reports indicate that integrase expression is regulated by the SOS system, that activates in response to DNA damage or to a stop in DNA replication, which leads to the accumulation of single strand DNA that in turn induces the expression of multiple genes related to recombination, repair and cell division (Foster, 2005; Erill et al., 2007). Our results suggest that streptomycin-induced stress leads to activation of the SOS system, which increases the expression of the integrase, resulting in the gene rearrangements observed in the plasmid-born integron. However, we could document cassette excision in both strains, which suggests that another type of regulation could be involved in the expression of the chromosomal *int*I1 gene of strain 3430-1. A higher *int*I1 gene dosage from the plasmid located integron could have contributed to the increased cassette rearrangements observed.

Not all Class 1 integrons are SOS-regulated since some of them contain a secondary promoter Pc2 that is activated by a GGG insertion that disrupts the *lex*A box, relieving the *int*I gene from SOS control (Cambray et al., 2010). Analysis of the upstream region of *int*I1 did not reveal a second promoter. Sequences also revealed identical *lex*A boxes in both strains, which contrasts with the lack of integrase induction in strain 3430-1. This apparent SOS independence would contribute to the production of basal levels of transcripts of antibiotic resistance genes, which would stabilize the integron in its chromosomal location.

## Conclusion

Our experiments show that identical integrons in two *Aeromonas* strains show a different behavior in response to streptomycin, which might result from differences in the host background.

## Author Contributions

All authors listed, have made substantial, direct and intellectual contribution to the work, and approved it for publication.

## Conflict of Interest Statement

The authors declare that the research was conducted in the absence of any commercial or financial relationships that could be construed as a potential conflict of interest. The reviewer PL-F and handling Editor declared their shared affiliation, and the handling Editor states that the process nevertheless met the standards of a fair and objective review.
